# Leishmanicidal Activity of Guanidine Derivatives against *Leishmania infantum*

**DOI:** 10.3390/tropicalmed8030141

**Published:** 2023-02-25

**Authors:** Fernanda Silva Almeida, Vitor Partite Moreira, Edson dos Santos Silva, Leonardo Lima Cardoso, Pedro Henrique de Sousa Palmeira, Luiz Henrique Agra Cavalcante-Silva, Demétrius A. M. de Araújo, Ian P. G. do Amaral, Eduardo René Pérez González, Tatjana S. L. Keesen

**Affiliations:** 1 Immunology of Infectious Diseases Laboratory of Department of Cellular and Molecular Biology; Federal University of Paraiba, João Pessoa 58051-900, PB, Brazil; 2 Biotechnology Doctoral Program, Rede Nordeste de Biotecnologia, Universidade Federal da Paraíba, João Pessoa 58051-900, PB, Brazil; 3 Department of Chemistry and Biochemistry, Fine Organic Chemistry Lab, School of Sciences and Technology, São Paulo State University (UNESP), Presidente Prudente 19060-080, SP, Brazil; 4 Department of Biotechnology, Universidade Federal da Paraíba, João Pessoa 58051-900, PB, Brazil; 5 Laboratory of Biochemistry, Department of Cellular and Molecular Biology; Federal University of Paraiba, João Pessoa 58051-900, PB, Brazil

**Keywords:** Leishmaniasis, anti-leishmanial activity, guanidine compounds, cell death, apoptosis, nitrite production

## Abstract

Leishmaniasis is a neglected tropical infectious disease with thousands of cases annually; it is of great concern to global health, particularly the most severe form, visceral leishmaniasis. Visceral leishmaniasis treatments are minimal and have severe adverse effects. As guanidine-bearing compounds have shown antimicrobial activity, we analyzed the cytotoxic effects of several guanidine-bearing compounds on *Leishmania infantum* in their promastigote and amastigote forms in vitro, their cytotoxicity in human cells, and their impact on reactive nitrogen species production. LQOFG-2, LQOFG-6, and LQOFG-7 had IC_50_ values of 12.7, 24.4, and 23.6 µM, respectively, in promastigotes. These compounds exhibited cytotoxicity in axenic amastigotes at 26.1, 21.1, and 18.6 µM, respectively. The compounds showed no apparent cytotoxicity in cells from healthy donors. To identify mechanisms of action, we evaluated cell death processes by annexin V and propidium iodide staining and nitrite production. Guanidine-containing compounds caused a significant percentage of death by apoptosis in amastigotes. Independent of *L. infantum* infection, LQOFG-7 increased nitrite production in peripheral blood mononuclear cells, which suggests a potential mechanism of action for this compound. Therefore, these data suggest that guanidine derivatives are potential anti-microbial molecules, and further research is needed to fully understand their mechanism of action, especially in anti-leishmanial studies.

## 1. Introduction

Leishmaniasis is a group of infectious and parasitic diseases caused by trypanosomatid protozoa of the genus *Leishmania* and is considered a neglected tropical disease. The three primary forms of the disease are cutaneous leishmaniasis (CL), visceral leishmaniasis (VL), also known as kala-azar, and mucocutaneous leishmaniasis (MCL). CL is the most common form, whereas VL is the most severe. More than 1 billion people live in areas endemic to leishmaniasis and are at risk of infection, with 30,000 new cases estimated annually [1,2].

*Leishmania donovani*, and *Leishmania infantum* cause VL. The anthroponotic form caused by *L. donovani* is prevalent in eastern Africa, Bangladesh, India, and Nepal, and *L. infantum* causes VL in the Mediterranean basin, China, the Middle East, and South America. It has the highest prevalence in Brazil, and its main reservoir is dogs [3]. Persistent irregular fever and splenomegaly characterize VL. Pancytopenia, hepatomegaly, hypergammaglobulinemia, and weight loss are common symptoms and are mainly present in the aggressive stages of the disease [4,5].

Successful treatment of leishmaniasis is challenging. For several years, leishmaniasis treatments have included pentavalent antimony, sodium stibogluconate, and meglumine antimoniate. However, in addition to painful intramuscular administration, antimonial is cardiotoxic, and its adverse effects are particularly evident in HIV-visceral leishmaniasis coinfection. Furthermore, sodium stibogluconate is no longer recommended in some countries owing to increased drug resistance [4,6].

Amphotericin B (AmB) has become the drug of choice in many countries. It binds to ergosterol, a sterol found in fungal and leishmanial but not in mammalian membranes. Its liposomal formulation, AmBisome, is safer than the other non-encapsulated drugs [5,6]. Miltefosine was adopted as a regimen by the Asian Elimination Initiative in 2005. However, *L. donovani* has grown resistant to this treatment, thus reducing its efficacy [4,7].

Organic compounds that contain guanidine groups can be found in nature, such as arginine and many natural alkaloids. Recently, interest in synthetic molecules and drugs containing guanidine moieties has increased [8]. Many compounds with a guanidine moiety have been synthesized in the last decade that show potent anti-microbial activity; some compounds have advanced to further drug developmental stages or clinical studies [8,9,10,11,12,13,14]. Due to their structural and molecular characteristics, guanidine compounds have been explored for treating infectious and non-infectious diseases. These compounds have shown potent activity against multiple pathogens, such as bacteria, by modulating antibiotic resistance in treatment-resistant *Staphylococcus aureus* [15], antifungal activity [9,16], antimalarial activity [17], and in vitro anti-parasitic activity against *L. infantum* and *Trypanosoma cruzi* [18].

In addition, because of the limitations of current treatments, the search for new drugs with leishmanicidal activity is critical [19]. Guanidine compounds have already shown activity in vitro and in vivo in BALB/c mice infected with *L. amazonensis* [11]. Therefore, in this study, we investigated the in vitro activity of several guanidine compounds against *L. infantum*, a strain that causes visceral leishmaniasis, to study their mechanisms of inducing cell death, cytotoxicity in human cells, and changes in reactive nitrogen species production.

## 2. Materials and Methods

### 2.1. Guanidine Compound Preparation

**Figure 1:**
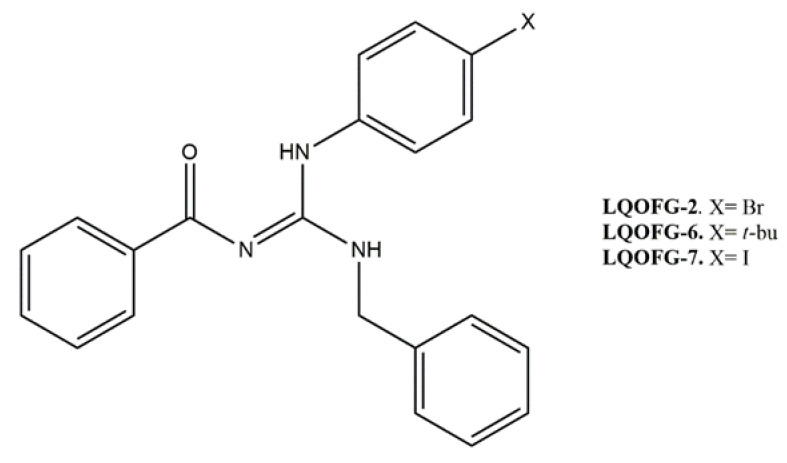
Molecular structure of guanidine derivatives. (*Z*)-N-((benzylamine)((4-bromophenyl)amino)methylene)benzamide (LQOFG-2). (*Z*)-N-((benzylamine)((4-(tert-butyl)phenyl)amino)methylene)benzamide (LQOFG-6). (*Z*)-N-((benzylamine)((4-iodophenyl)amino)methylene)benzamide (LQOFG-7) [11].

Three guanidine compounds (Figure 1) were synthesized according to the protocol described by Espírito Santo and collaborators [11] and diluted in dimethyl sulfoxide (DMSO) to obtain stock solutions (20 mg/mL). To obtain the final concentrations of the drugs in the assays, the stock solutions were diluted in a culture medium with a concentration of up to 0.5% DMSO (vehicle control).

### 2.2. Reference Anti-L. infantum Drug

Amphotericin B (Anfotericin B^®^; Cristália, São Paulo, Brazil) was used as a positive control, as it is an anti-*Leishmania* drug. A stock solution (10 mg/mL) was prepared in DMSO.

### 2.3. L. infantum Culture Conditions

The promastigote forms of *Leishmania* (*Leishmania*) *infantum* [IOC/L0579(MHOM/BR/1974/PP75)] were cultured in Schneider’s medium, pH 7.0, supplemented with 20% heat-inactivated fetal bovine serum (FBS), 2% male human urine, 100 U/mL of penicillin, and 100 mg/L of streptomycin, and the parasites were maintained at 26 °C. The procedure to obtain the extracellular axenic amastigote form of *L. infantum* was based on methods previously described [20] that were modified using a Schneider medium readjusted to pH 5.5 at 37 °C. The culture of promastigote forms in the stationary phase was centrifuged, and the medium of these cells was replaced and differentiated into axenic amastigotes by temperature and medium pH changes. Parasites were maintained in culture for no more than 20 passages.

### 2.4. Anti-L. infantum Activity

The promastigote growth inhibition assay was performed as previously described [21]. Promastigotes were harvested during the exponential growth phase. They were incubated in the presence and absence of several concentrations of LQOFG-2, LQOFG-6, LQOFG-7 (500 to 7.8 µM), and AmB (10 to 0.078 µM) as the positive control. The plate was incubated at 26 °C for 72 h in a biological oxygen demand (B.O.D.) incubator using a Schneider’s medium (Schneider’s Insect Medium 24.5 g/L; L-glutamine 1.8 g/L; glucose 2 g/L and sodium bicarbonate 0.4 g/L; Sigma-Aldrich—St. Louis—USA) supplemented. The growth inhibition was evaluated using an MTT assay kit (Amresco, Solon, OH, USA) according to the manufacturer’s protocol. After 4 h of incubation, 10% sodium dodecyl sulfate was added to dissolve the formazan crystals. The absorbance (540 nm) was measured using a plate reader (Biosystems model ELx800; Curitiba, PR, Brazil). The same procedure was followed to assess inhibition of the axenic amastigote form using these compounds with the following modifications: the treatment time was shortened to 24 h, and the test temperature was raised to 37 °C at a pH of 5.5. Three independent experiments we performed in triplicate.

### 2.5. Red Blood Cell Lysis Assay

The hemolytic activities of guanidine compounds were measured using human red blood cells from healthy adults (*n* = 3) according to methods previously described [22]. Briefly, 80 µL of a 5% erythrocyte/phosphate-buffered saline (PBS) suspension was mixed with 20 µL of LQOFG-2, LQOFG-6, LQOFG-7, (1000 to 7.8 µM), and AmB (100 to 0.78 µM). After incubation at 37 °C for 1 h, 200 µL of PBS (1.5 mM KH_2_PO_4_, 8.1 mM Na_2_HPO_4_, 136.9 mM NaCl, and 2.6 mM KCl, pH 7.2) was added to stop the hemolysis process, and the samples were centrifuged for 10 min at 1000× *g*. The supernatants were collected, and the extent of hemolysis was measured spectrophotometrically at 540 nm. The hemolysis percentage was determined as [(Abs_sam_ − Abs_con_)/(Abs_tot_ − Abs_con_) × 100], where Abs_sam_ is the absorbance of the sample, Abs_con_ is the absorbance of the blank control (without drugs), and Abs_tot_ is the absorbance of total hemolysis (replacing the sample solution with an equal volume of ultrapure water (Direct-Q UV^®^, Guyancourt, France)).

### 2.6. In Vitro Cytotoxicity in Peripheral Blood Mononuclear Cells 

In vitro cytotoxicity was evaluated using peripheral blood mononuclear cells (PBMCs) collected from three healthy volunteers (2 males, mean age 33; and 1 female, age 28). PBMCs were separated by density gradient using Ficoll Paque PLUS (GE Healthcare, USA), pelleted by centrifugation (400*× g* for 40 min), and resuspended in RPMI 1640 medium, then supplemented with 10% fetal bovine serum and 1% antibiotic solution (penicillin 5000 Units/mL + streptomycin 5000 μg/mL). After 24 h of treatment, cell viability was evaluated by the modified colorimetric method based on the tetrazolium dye MTT (3-(4,5-dimethylthiazol-2-yl)-2,5- diphenyltetrazolium bromide). The MTT assay was used for in vitro cytotoxicity as previously described with the following modifications: cells were exposed to serially diluted concentrations of LQOFG-2, LQOFG-6, LQOFG-7 (1000 to 7.8 µM), and AmB (100 to 0.78 µM). The plates were kept at 37 °C for 24 h. MTT (5 mg/mL) was added and incubated for 4 h at 37 °C, after which the plates were centrifuged, the supernatant was discarded, and DMSO was added, leaving the plates under stirring to solubilize the formazan salts. Absorbance was measured at 540 nm using a plate reader (ELx800; Curitiba, PR, Brazil). The mean percentage of viable cells was calculated relative to the untreated control.

### 2.7. Apoptosis/Necrosis Profiling with Annexin V/PI Staining in Promastigote and Amastigote-Like Forms

To evaluate both apoptosis and necrosis, promastigote forms of *L. infantum* in the log phase of growth were incubated at several concentrations based on the IC_50_ values of LQOFG-2 (6.35; 12.7, 25.4, and 50.8 µM) LQOFG-6 (12.2, 24.4, 48.8 and 97.6 µM), and LQOFG-7 (11.65, 23.3, 46.6 and 93.2 µM), and AmB (1.5 and 6 µM) was used as the positive control. The plates were incubated for 24 h at 26 °C ± 1 °C in a B.O.D. incubator. After incubation, the promastigotes were washed three times in cold PBS, resuspended in binding buffer (10-mM HEPES, 140-mM NaCl, and 2.5-mM CaCl2, pH 7.4), and stained using a FITC Annexin V/Dead Cell Apoptosis Kit (BD Biosciences, San Jose, CA, USA) according to the manufacturer’s instructions. The stained cells were diluted and suspended in an Annexin V binding buffer (BD Biosciences, San Jose, CA, USA). Annexin V-FITC/propidium iodide (PI)-stained cells were analyzed using a BD FACSCanto II flow cytometer (BD Biosciences, San Jose, CA, USA). The raw data were analyzed using FlowJo 10.0.7 software (TreeStar Inc., Ashland, OR, USA).

To evaluate both apoptosis and necrosis of the amastigote-like forms of *L. infantum*, the protocol was followed. In each well, amastigotes were treated with the guanidine compounds and controls. Several concentrations based on the EC_50_ values of LQOFG-2 (13, 26.1, 52.2, and 104.4 µM), LQOFG-6 (10.5, 21.1, 42.2, and 84.4 µM), and LQOFG-7 (9.3, 18.6, 37.2 and 72.4 µM) were used, and AmB (0.30 and 1.2 µM) was used as the positive control.

### 2.8. Nitrite Assay

To perform the nitrite assay, PBMCs were obtained from five healthy volunteers (3 males, mean age 28.67 ± 2.96; and 2 females, mean age 28.50 ± 0.5) from João Pessoa, PB Brazil. Briefly, peripheral blood was diluted 1:1 with phosphate-buffered saline (PBS) and slowly layered over using Ficoll^®^ Paque PLUS (GE Healthcare, Waukesha, WI, USA). Tubes were centrifuged at 400× g for 40 min at 20 °C. After centrifugation, PBMCs were harvested, washed three times with PBS, and resuspended in RPMI medium supplemented with antibiotics (penicillin 200 U/mL and streptomycin 0.1 mg/mL), 1 mM l-glutamine, and 10% inactivated human serum. Cell viability was assessed using a trypan blue dye exclusion assay. Cells were counted using a hemocytometer. [23,24]

Promastigotes were prepared as previously described [24]. Briefly, to confirm the infection, the *L. infantum* promastigotes were labeled with carboxyfluorescein diacetate succinimidyl ester (CFSE) (CellTrace ™ CFSE Cell Proliferation Kit; Eugene, OR, USA). The suspension was incubated for 15 min at 5% CO_2_, 37 °C, and washed three times with PBS containing 10% inactivated FBS producing CFSE-labeled *L. infantum*, which was added to PBMCs. The infection ratio was set at 1:10 (monocyte: *L. infantum*) for 3 h. After the infection, cells were washed three times with PBS supplemented with 10% FBS.

The plates were incubated for 24 h in a 5% CO_2_ incubator at 37 °C. The plates were centrifuged at 200× *g* for 8 min at 4 °C, and the supernatant was collected to measure reactive nitrogen production. CD14 surface antibody (PERCP-Cy5.5; clone MΦP9) was used for monocyte labeling (Figure S1). After adding the antibody, the plate was incubated at 4 °C for 15 min, protected from light. The cells were washed with ice-cold PBS buffer. The plate was centrifuged at 200× *g* again for 10 min at 4 °C. The supernatant was removed, the cells were resuspended in 100 µL of PBS buffer and 100 µL of 4% formaldehyde solution, then read on a flow cytometer (BD FACSCanto II, BD Biosciences, San Jose, CA, USA). FlowJo v.10.8.1 software was used to analyze the raw data [25]. After obtaining PBMC, the cells were plated and incubated with LQOFG-7 and AmB at a concentration of 1× the EC_50_ value, with media or lipopolysaccharide (LPS) stimulus (lipopolysaccharide derived from Escherichia coli, Sigma-Aldrich O111:B4) at 100 ng/mL [26,27,28]. The culture supernatants from those infected and non-infected were evaluated for the stable end-product of reactive nitrogen species (NO, nitrates, and nitrites) using the Griess reaction, according to the Cayman Chemical instruction manual (Cayman Chemical, Ann Arbor, MI, USA). The nitrite level was determined by measuring the absorbance at a wavelength of 540 nm using a microplate reader (BioTek-ELx800) [29].

### 2.9. Data Analysis and Statistics

The 50% inhibitory concentration (IC_50_), 50% effective concentration (EC_50_), 50% cytotoxic concentration (CC_50_), and 50% hemolytic concentration (HC_50_) values were calculated using the software GraphPadPrism^®^ program (version 6.0; San Diego, CA, USA). Statistical analysis was performed using nonlinear regression (curve fit). Unless otherwise noted, the assays were performed in triplicates and three independent experiments. We used FlowJo 10.0.7 for the Flow cytometry data analysis. Statistical differences among treatments were assessed by analysis of variance (ANOVA) with the post hoc Tukey test at a significance level of 0.05. The data represent the mean ± standard error (SEM).

### 2.10. Ethics Statement

All experiments complied with the relevant laws, institutional guidelines, and ethical standards of the Declaration of Helsinki. Furthermore, written informed consent was obtained from all healthy volunteers, and the Ethics Committee approved the study at the Federal University of Paraiba, Brazil (process number: 2.560.067 and CAAE:82944118.5.0000.5188).

## 3. Results

### 3.1. In Vitro Antileishmanial Activity and Selectivity Index Calculation

The half-maximal inhibitory concentrations (IC_50_) of the growth of the *L. infantum* promastigotes of LQOFG-2, LQOFG-6, and LQOFG-7 were 12.7 µM, 24.4 µM, and 23.6 µM, while the IC_50_ for Amphotericin B was 1.5 µM (Table 1; Figure S2).

At the highest concentration tested (1000 µM), none of the guanidine-containing compounds were toxic to human red blood cells; therefore, it was not possible to calculate the selectivity index (the ratio HC_50_/IC_50_). However, AmB presented an HC_50_ of 7.10 ± 2.09 µM, producing a selectivity index of 4.73, which matches the already known cellular toxicity of AmB [30].

**Figure 2:**
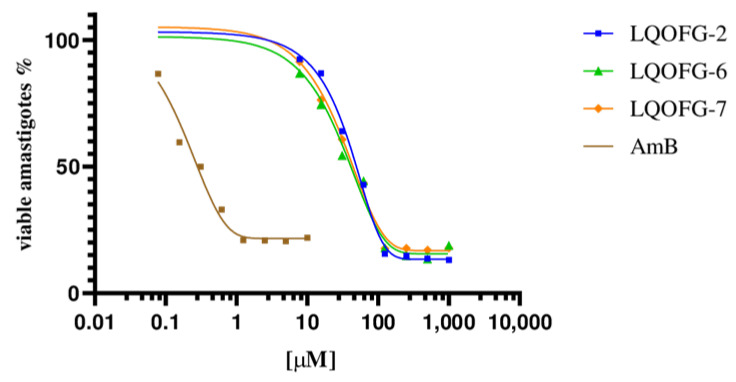
Cell viability % of axenic amastigotes treated with LQOFG-2, LQOFG-6, LQOFG-7, and Amphotericin B.

The LQOFG-2, LQOFG-6, and LQOFG-7 showed cytotoxic activity in axenic amastigotes with EC_50_ values of 26.1 ± 1.22 µM, 21.1 ± 1.13 µM, and 18.6 ± 1.23 µM, respectively. The AmB had an EC_50_ value of 0.30 ±1.29 µM (Table 2; Figure 2). At the concentration of 31.2 µM, the LQOFG-2, LQOFG-6, and LQOFG-7 presented inhibitions of 57.71%, 59.88%, and 58.02%, respectively, compared to the control (untreated).

**Figure 3:**
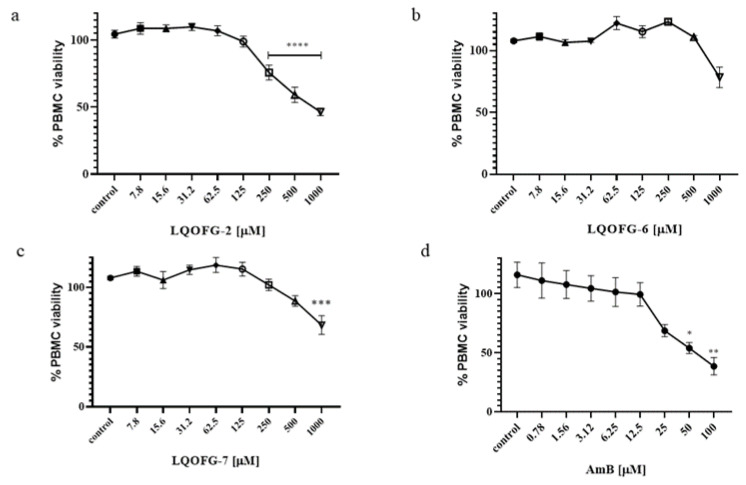
PBMC % viability after LQOFG-2 (**a**), LQOFG-6 (**b**), LQOFG-7 (**c**), and AmB (**d**) treatment. Data are mean ± SEM from three independent experiments in triplicate. (control): represents untreated control. * *p* ≤ 0.05, ** *p* ≤ 0.01, *** *p* ≤ 0.001, **** *p* ≤ 0.0001 by analysis of variance (ANOVA) with the post hoc Tukey test.

The LQOFG-2, LQOFG-6, and LQOFG-7 had a CC_50_ of 313.1 ± 3.23, > 1000, and 745.5 ± 3.58, respectively, while Amphotericin B had a CC_50_ of 53.88 ± 3.59 (Table 2; Figure 3; Figure S3). The AmB showed more significant toxicity; however, its lower EC50 had a better selectivity index than guanidine compounds. In contrast, the compound LQOFG-2 had the highest toxicity to PBMC among the guanidine derivatives, but it was less toxic than the AmB.

### 3.2. Apoptosis/Necrosis Profiling in Promastigote and Amastigote-Like Forms

Flow cytometry with annexin V-FITC and PI staining was used to identify cell death stages and pathways in parasites after treatment with LQOFG-2, LQOFG-6, and LQOFG-7. Annexin V and PI discriminate between early- (AV+, PI−) and late-apoptotic cells (AV+, PI+) as well as necrotic (AV−, PI+) and live cells (AV–, PI−) [31,32]. 

**Figure 4:**
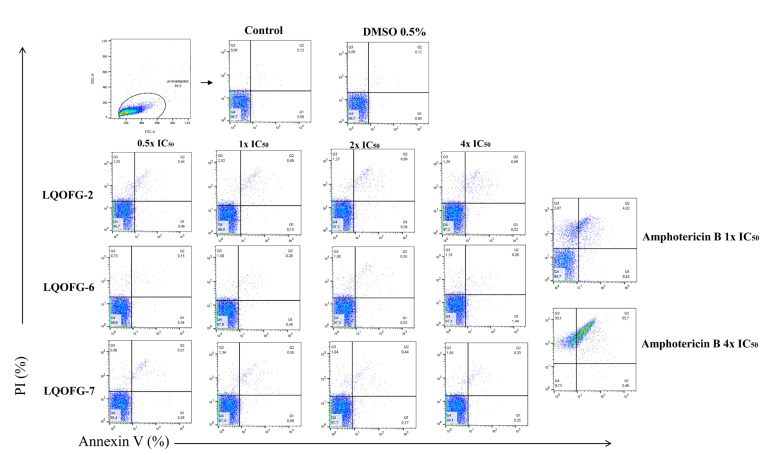
Analysis strategy of the representative dot plots showing staining of *L. infantum* promastigotes. *L. infantum* promastigotes were incubated at 26 °C for 24 h in the absence or presence of the LQOFG-2, LQOFG-6, LQOFG-7, or AmB at concentrations of 0.5×, 1×, 2×, or 4× the IC_50_. Annexin V/ PI (propidium iodide) staining was performed, and the samples were analyzed by flow cytometry. Q1: early apoptosis (AV+, PI−); Q2: late apoptosis (AV+, PI+); Q3: necrotic cells (AV−, PI+); Q4: live cells (AV−, PI−).

There were no statistically significant changes for the guanidine compounds. Amphotericin B induced late-apoptosis at the rates of 4.30 ± 0.28% at 1× the IC_50_ and 65.83 ± 1.85% at 4× the IC_50_. The necrotic cell percentage (AV−, PI+) was 7.64 ± 1.17% at 1× the IC_50_ and 33.10 ± 1.90% at 4× the IC_50_ concentrations (*p* < 0.0001, Table S1; Figure 4).

**Figure 5:**
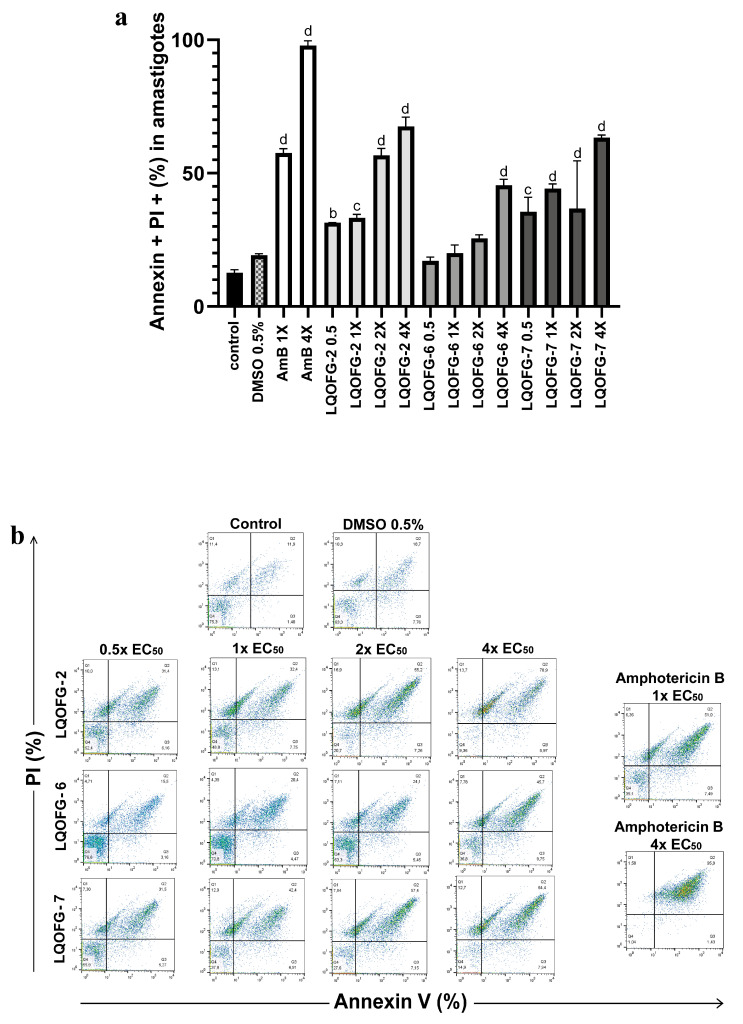
Cell death of axenic amastigotes following LQOFG-2, LQOFG-6, LQOFG-7, and AmB treatment. (**a**) Percentage of cell death by late apoptosis of axenic amastigotes. (**b**) Representative dot plots showing staining of *L. infantum* axenic amastigotes. Axenic amastigotes were incubated in the absence or presence of LQOFG-2, LQOFG-6, LQOFG-7, or AmB at concentrations of 0.5×, 1×, 2×, or 4× the EC_50_. Annexin V/PI (propidium iodide) staining was performed, and the samples were analyzed by flow cytometry. ^b^ *p* ≤ 0.01 vs. untreated control, ^c^ *p* ≤ 0.001 vs. untreated control, ^d^ *p* ≤ 0.0001 vs. untreated control, by analysis of variance (ANOVA) with the post hoc Tukey test compared to the untreated control.

The same assay parameters were used for the axenic amastigotes of *L. infantum* in the stationary phase, which included 24 h of treatment at 0.5×, 1×, 2×, and 4× the EC_50_. The data for these experiments can be found in Table 3 and Figure 5. 

There was no statistical difference between the negative control (untreated) and vehicle control (DMSO 0.5%). The LQOFG-2 showed statistically significant results in late apoptosis (AV+, PI+) at all tested concentrations. For the LQOFG-6, early and late apoptosis (*p* < 0.0001) were statistically significant at 4× the EC_50_. The LQOFG-7 treatment produced statistically significant cell death (*p* < 0.05 to *p* < 0.0001) at all tested concentrations (Figure 5). Only the AmB at 4× the EC_50_ showed a significant difference in the necrotic profile (AV–, PI+) compared to the control. The AmB showed a percentage of late apoptosis of 57.50 ± 1.72% at 1× the EC_50_ concentration and 97.87 ± 1.01% at 4× the EC_50_.

### 3.3. Nitrite Levels from PBMC after L. infantum Infection

As the LQOFG-7 was the most active compound based on its anti-Leishmanial activity, low toxicity in human PBMCs, and ability to induce death by apoptosis in *L. infantum* amastigotes, we selected this compound to investigate whether guanidine-containing compounds could affect infectivity and the production of reactive nitrogen species.

We investigated whether the LQOFG-7 could increase reactive nitrogen species production in human monocytes. A significant increase in nitrite production can be seen in monocytes treated with LQOFG-7 (10.5 ± 3.58 µM) compared with the untreated control group (3.81 ± 1.36 µM) (*p* < 0.05). There was no significant difference between the LPS-treated cells and the LQOFG-7 treatment under both conditions. This assay found that the guanidine derivative LQOFG-7 induced nitrite production in PBMCs.

**Figure 6:**
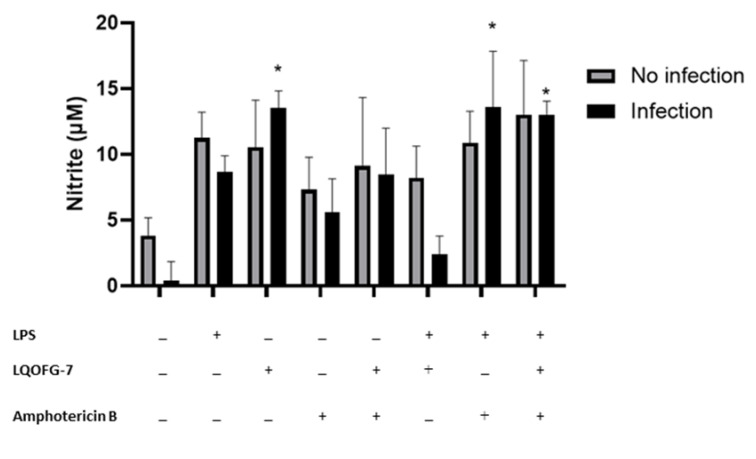
Nitrite levels are produced by monocytes infected with *L. infantum* treated with LQOFG-7, AmB, or LPS. The Griess reaction measured nitrite levels from supernatants collected from the *L. infantum* monocyte infection rate assay in PBMCs. Cells were either infected or not infected. * *p* < 0.05. The graph represents the mean ± SEM. One-way ANOVA, followed by post hoc Tukey’s test, was performed to compare groups with the respective untreated controls.

Little additional nitrite was produced by monocytes infected with *L. infantum* (0.367 ± 1.48 µM) (Figure 6). LPS-activated monocytes produced nitrite at a concentration of 8.67 ± 1.23 µM. The LQOFG-7 treatment significantly increased nitrite production (13.5 ± 1.30 µM) in inactivated monocytes (*p* < 0.05). There was a decrease in nitrite production (2.43 ±1.36 µM) in LPS-activated-infected monocytes treated with LQOFG-7 compared to uninfected monocytes. Statistical data analysis showed no differences in nitrite production between infected monocytes treated with LQOFG-7 and the LPS control.

We also evaluated the effect of AmB treatment (EC_50_) on nitrite production. There was no significant increase in nitrite production in infected monocytes treated with AmB. However, LPS-activated monocytes exhibited a substantial increase in nitrite production (13.6 ± 4.25 µM) (*p* < 0.05) compared to untreated infected cells (0.367 ± 1.48 µM). In addition, we evaluated whether there was a combination effect between the AmB and LQOFG-7 treatment. We found an increase in nitrite production (*p* < 0.05; 13.0 ± 1.04 µM) in the LPS-activated monocytes.

## 4. Discussion

Previous studies on the anti-Leishmanial activity of guanidine derivatives have yielded favorable results [8,13]. Of the guanidine compounds, the LQOFG-2 reduced promastigote cell viability in the MTT assay the most. Previous reports indicated that LQOFG-2 (-Br) and LQOFG-7 (-I) had better anti-Leishmanial activity against *Leishmania amazonensis* intracellular amastigote forms [11]. The anti-promastigote data (IC_50_ data, Table 1) suggested that the substituent -Br may contribute to this action. Previous research has shown that halogen substituents, such as -Cl and -Br, show promising effects in this area [15]. The compounds we studied did not show toxicity to human red blood cells and should be the focus of future preclinical studies. The AmB showed cytotoxicity in healthy human donor red blood cells at 7.10 ± 2.09 µM. This drug is used to treat leishmaniasis and has limitations, such as nephrotoxicity and other adverse effects, which warrant the development of new medicines against leishmaniasis [7,30].

Guanidine-bearing compounds are more toxic to the amastigotes rather than the promastigotes. The LQOFG-7 derivative presented a better cytotoxic activity profile at micromolar values (18.6 µM) with PBMC safety. However, the cytotoxic activities of the three compounds against amastigotes were similar. Their substituents, halogens, and a *t-*butyl group (Figure 1) favor cytotoxic activity with positive lipophilic effects [33].

In the present study, the guanidine compounds had low toxicity to human PBMCs with high selectivity indices for the LQOFG-6 and LQOFG-7. On the other hand, the AmB was more cytotoxic to red blood cells and PBMCs but had a higher selectivity index owing to its low EC_50_ value. However, previous research on anti-Leishmanial drugs on primary human immune cells reported that 20 μM of AmB induced cell death by mitochondrial membrane depolarization, which was indicated by phosphatidylserine exposure [34].

Significant changes occur in the plasma membrane during cell death. Phosphatidylserine (PS) externalization is one of the main changes in the plasma membrane during apoptosis, because cells identify PS as a marker for needed phagocytosis [32,35,36]. In early apoptosis, the plasma membrane integrity excludes PI; therefore, annexin V staining is a marker of early apoptosis. PS exposure is a good marker for apoptosis. However, because *Leishmania* spp. use apoptotic mimicry to invade phagocytic cells, studying cell death mechanisms in pathogenic organisms is essential. In amastigotes, apoptotic mimicry is the ability to expose PS without death; this ability is critical, as exposed PS plays a role in phagocytosis cell recognition and engulfment, which has been observed in vivo [32,36,37] Through this process, parasites, especially amastigotes, inhibit host macrophage pro-inflammatory responses and induce anti-inflammatory cytokines to allow for parasite proliferation and disease progression [37,38,39]. In contrast to the in vivo data, our in vitro data demonstrated that the guanidine compounds induce cell death by apoptosis in amastigote-like forms of *L. infantum*.

Different toxicity outcomes are possible in other protozoan life stages, with an increase, decrease, or even loss of harmful activity. For example, Martins et al. [18] studied the in vitro antiparasitic activity of synthetic analogs of guanidine alkaloids from a marine sponge against *L. infantum* and *T. cruzi*. They reported that some compounds had the highest selectivity index for the amastigote forms of *L. infantum*. Our results showed no statistically significant effect of guanidine derivatives on cell death in promastigotes. In contrast, the AmB demonstrated an apparent apoptotic impact similar to that observed in previous studies [40,41]. However, the LQOFG-2 and LQOFG-6 treatments resulted in a gradual increase in late-apoptotic axenic amastigotes cells. These results indicate that increasing drug concentrations significantly affect parasites (Table 3; Figure 3).

AmB induced significant levels of necrosis in all the assays; of note, the main target of the AmB is the cell membrane sterol ergosterol, which results in the loss of cell barrier protection [42,43]. Guanidine compounds induced early apoptosis (AV+, PI−) in amastigote forms at similar levels to vehicle control. *L. infantum* axenic amastigotes cell death was induced by the guanidine compounds. There have been no studies on how guanidine compounds induce apoptosis in cells. However, other researchers have reported that guanidine analogs affect mitochondrial membrane potential depolarization and increase the levels of reactive oxygen species, as well as plasma membrane permeability, in *Leishmania* parasites [18].

To investigate the possible immunomodulatory mechanisms of LQOFG-7, nitrite production was evaluated in human PBMCs following infection with *L. infantum*. Reactive nitrogen species levels are essential, as these species control pathogen infection, mainly through intracellular amastigotes in leishmaniasis. Furthermore, nitric oxide synthase (NOS) expression and NO production are characteristic of immune cell responses; in particular, innate immune cells are essential sources of NO, mainly in dendritic cells and monocytes/macrophages, but also in natural killer cells, eosinophils, mast cells, and neutrophils [44,45].

In our study (Figure 6), *L. infantum* infection in monocytes decreased nitrite production in the absence of treatment, which is consistent with previous reports [38]. Previous researchers studied PBMCs from patients with visceral leishmaniasis and reported reduced NO production and higher expression of IL-10 and TGF-β [46]. These cytokines have been noted to be involved in how *Leishmania* evades host macrophages.

After treating infected monocytes with LQOFG-7 at the EC_50_ value, NO production was significantly increased. Adding LPS stimulus with LQOFG-7 treatment did not affect nitrite production. We also observed a slight increase in NO production in uninfected monocytes after treatment with LQOFG-7. No studies have reported this activity of guanidine-containing compounds in leishmaniasis. Combining chemo- and immunotherapy is one strategy that may be beneficial for intracellular infections, such as leishmaniasis [47]. In addition to its well-established role as an anti-microbial effector mechanism, NO, produced by inducible NO synthase (iNOS), provides critical immunomodulatory feedback [48]. NO is a crucial inter- and intracellular messenger molecule that maintains vascular tone, neuronal signaling, and the host response to infection [49].

In VL infection, the phagosome membrane is an additional barrier that prevents drugs from entering macrophages. Amastigotes residing in phagosomes also interfere with host defense mechanisms by inhibiting macrophage iNOS as part of its survival strategy [50]. Cytokines, such as IFN-γ, induce macrophages to produce NO, which can eliminate these intracellular amastigotes. A group evaluated the expression of TLR2 and TLR4 receptors with cytokine and NO production in the PBMCs of patients with VL before and after treatment with meglumine antimoniate and AmB. They found that non-stimulated cells produced significantly lower NO levels than the cells post-treatment, indicating lower levels of phagocyte-residing parasites [51]. LPS stimulation increased NO levels compared to unstimulated cells in pre- and post-treatment patients and control individuals. Here, we found that nitrite production increased in infected cells after treatment with AmB, LPS stimulation, and LPS + LQOFG-7 (*p* < 0.05) (Figure 6).

Considering the present limits in treating leishmaniasis, several studies have sought immunological strategies to prevent and treat leishmaniasis. In one attempt, immunotherapy alone or with chemotherapy was developed to treat leishmaniasis infection and avoid the side effects of conventional treatment regimens. Immunotherapy aims to accelerate the patient’s targeted and specific response to the parasite, and this treatment results in effective reactions in patients without using conventional drugs [47].

In conclusion, visceral leishmaniasis is a neglected tropical disease, and the current treatments have severe adverse effects. Furthermore, new, effective, and accessible therapies are limited. In the present study, we demonstrated that the guanidine-bearing compound, LQOFG-7, had the best activity profile against *L. infantum*, featuring a good selectivity index and low cytotoxicity in PBMCs, and it is capable of inducing apoptosis in amastigotes. However, further studies are required to characterize LQOFG-7 in an environment with an effector or immunological response.

## Figures and Tables

**Table 1:** The half-maximal inhibitory concentration of the growth of *L. infantum* promastigotes (IC_50_) treated with guanidine-derived compounds and controls.

Compounds	IC_50_ (µM)	HC_50_ (µM)	SI
LQOFG-2	12.7 ± 0.25	>1000	>78.2
LQOFG-6	24.4 ± 0.76	>1000	>40.98
LQOFG-7	23.6 ± 0.64	>1000	>42.37
Amphotericin B	1.5 ± 0.23	7.10 ± 2.09	4.73

The data represent mean ± SEM from three independent experiments in triplicate of *L. infantum* promastigotes (IC_50_) treated with guanidine-derived compounds and amphotericin B as a positive control for 72 h of treatment, toxicity to red blood cells (HC_50_), and the selectivity indices (SI, HC_50_/IC_50_). Nd = not determined.

**Table 2:** Half-maximal effective concentration (EC_50_) of axenic amastigotes treated with LQOFG-2, LQOFG-6, LQOFG-7, and AmB as the positive control, cytotoxicity in PBMC cells (CC_50_), and the selectivity index (SI, CC_50_/EC_50_).

Compounds	EC_50_ (µM)	CC_50_ (µM)	SI
LQOFG-2	26.1± 1.22	313.1 ± 3.23	12.00
LQOFG-6	21.1 ± 1.13	>1000	>47.4
LQOFG-7	18.6 ± 1.23	745.5 ± 3.58	39.54
Amphotericin B	0.30 ± 1.29	53.8 ± 3.59	177.6

The data represent mean ± SEM from three independent experiments in triplicate. Nd = not determined.

**Table 3:** Percentage of cell death by apoptosis/necrosis of *L*. *infantum* axenic amastigotes treated with LQOFG-2, LQOFG-6, LQOFG-7, or AmB for 24 h.

% Cells (Amastigote)			
Early Apoptosis (AV+, PI−)		Late Apoptosis (AV+, PI+)	Necrosis (AV−, PI+)
Cells	1.99 ± 0.29	12.63 ± 0.68	15.57 ± 0.33
DMSO 0.5%	3.99 ± 0.48	19.20 ± 0.55	13.00 ± 2.26
LQOFG-2 0.5 × EC_50_	5.68 ± 0.29 ^a^	31.43 ± 0.03 ^b^	13.20 ± 1.62
LQOFG-2 1 × EC_50_	8.13 ± 0.84 ^d^	33.23 ± 0.73 ^c^	11.97 ± 0.66
LQOFG-2 2 × EC_50_	7.68 ± 0.39 ^d^	56.27 ± 1.51 ^d^	15.93 ± 1.02
LQOFG-2 4 × EC_50_	7.55 ± 1.18 ^d^	67.50 ± 2.05 ^d^	13.80 ± 1.47
LQOFG-6 0.5 × EC_50_	3.62 ± 0.39	17.10 ± 0.80	13.20 ± 1.62
LQOFG-6 1 × EC_50_	4.05 ± 0.22	20.00 ± 1.74	6.57 ± 1.20
LQOFG-6 2 × EC_50_	5.72 ± 0.20 ^a^	25.50 ± 0.80	7.15 ± 0.21
LQOFG-6 4 × EC_50_	9.06 ± 0.70 ^d^	45.47 ± 1.30 ^d^	8.14 ± 0.78
LQOFG-7 0.5 × EC_50_	5.56 ± 0.46 ^a^	35.53 ± 3.13 ^c^	9.83 ± 1.27
LQOFG-7 1 × EC_50_	5.84 ± 0.57 ^b^	44.20 ± 1.01 ^d^	13.53 ± 0.32
LQOFG-7 2 × EC_50_	10.02 ± 1.53 ^d^	36.70 ± 10.35 ^d^	5.63 ± 1.24
LQOFG-7 4 × EC_50_	8.01 ± 0.14 ^d^	63.27 ± 0.59 ^d^	12.27 ± 0.48
Amphotericin B 1 × EC_50_	4.73 ± 0.97	57.50 ± 1.72 ^d^	11.52 ± 1.89
Amphotericin B 4 × EC_50_	0.60 ± 0.42	97.87 ± 1.01 ^d^	0.96 ± 0.33 ^d^

Percentage of cell death by apoptosis/necrosis of axenic amastigote forms ± SEM from three independent experiments in triplicate. ^a^ *p* < 0.05 vs. untreated control, ^b^ *p* < 0.01 vs. untreated control, ^c^ *p* < 0.001 vs. untreated control, ^d^ *p* < 0.0001 vs. untreated control, by analysis of variance (ANOVA) with the post hoc Tukey test, compared with the untreated control.

## Data Availability

All data supporting the study findings are included in this published article or supplementary material.

## Supplementary Materials

The following supporting information can be downloaded at: https://www.mdpi.com/article/10.3390/tropicalmed8030141/s1, Figure S1: Analysis strategy of monocytes derived from human peripheral blood mononuclear cell (PBMC) using carboxyfluorescein diacetate succinimidyl ester (CFSE)-labeled *Leishmania infantum*. The PBMC cells were visualized on FSC vs. SSC and a wide gate was performed around the monocyte population, excluding most debris and lymphocytes. The cells were visualized on the FSC-H x FSC-A plot in single cells format. These cells were then visualized on the CD14 (PERCP) vs granularity (SSC) plot by selecting only the monocyte population. Finally, CD14+ cells were visualized on the CFSE vs. plot. CD14+, thus identifying monocytes infected by *Leishmania* sp; Figure S2: Dose-response curve fitting of promastigotes treated with LQOFG-2; LQOFG-6; LQOFG-7 and Amphotericin B; Figure S3: Dose-response curve fitting of PBMC cytotoxicity after LQOFG-2, LQOFG-6, LQOFG-7, and Amphotericin B treatment; Table S1: Percentage of cell death by apoptosis/necrosis of promastigote forms of *Leishmania infantum* treated with guanidine derivatives and the reference drug amphotericin B for 24h.
