# Supplementary material for: Leishmanicidal Activity of Guanidine Derivatives against Leishmania infantum
Source: Trop Med Infect Dis. 2023 Feb 25;8(3):141. doi: 10.3390/tropicalmed8030141 (PMC10051705; doi:10.3390/tropicalmed8030141)
Supplement: Supplementary file 1 [file tropicalmed-08-00141-s001.zip › tropicalmed-2125185-supplementary.pdf]

Tropical Medicine and Infectious Disease

MDPI

Supplementary Materials: Leishmanicidal Activity of Guanidine Derivatives against *Leishmania infantum*

Table S1. Percentage of cell death by apoptosis/necrosis of promastigote forms of *Leishmania infantum* treated with guanidine derivatives and the reference drug amphotericin B for 24h.

	% Cells (Promastigote)		
	**Early Apoptosis (AV+, PI−)**	**Late Apoptosis (AV+, PI+)**	**Necrosis (AV− PI+)**
**Control**	0.89 ± 0.15	0.25 ± 0.07	0.71 ± 0.09
**DMSO 0.5%**	0.62 ± 0.06	0.60 ± 0.08	0.83 ± 0.01
**LQOFG-2 0.5 ×IC_{50}**	0.27± 0.09	0.55 ± 0.04	1.69 ± 0.28
**LQOFG-2 1× IC_{50}**	0.12 ± 0.02[a]	0.41 ± 0.14	2.07 ± 0.23
**LQOFG-2 2× IC_{50}**	0.35 ± 0.01	0.78 ± 0.15	1.52 ± 0.24
**LQOFG-2 4× IC_{50}**	0.34 ± 0.09	0.63 ± 0.12	1.79 ± 0.59
**LQOFG-6 0.5× IC_{50}**	0.65 ± 0.19	0.18 ± 0.01	0.85 ± 0.09
**LQOFG-6 1× IC_{50}**	0.46 ± 0.13	0.20 ± 0.03	1.47 ± 0.21
**LQOFG-6 2× IC_{50}**	0.51 ± 0.08	0.32 ± 0.06	1.37 ± 0.22
**LQOFG-6 4× IC_{50}**	0.91 ± 0.34	0.20 ± 0.03	1.06 ± 0.07
**LQOFG-7 0.5× IC_{50}**	0.28 ± 0.05	0.47 ± 0.07	0.87 ± 0.17
**LQOFG-7 1× IC_{50}**	0.77 ± 0.04	0.41 ± 0.08	1.19 ± 0.12
**LQOFG-7 2× IC_{50}**	0.76 ± 0.10	0.26 ± 0.09	1.37 ± 0.22
**LQOFG-7 4× IC_{50}**	0.39 ± 0.08	0.18 ± 0.07	1.06 ± 0.07
**Amphotericin B 1× IC_{50}**	0.67 ± 0.27	4.30 ± 0.28[d]	7.64 ± 1.17[d]
**Amphotericin B 4× IC_{50}**	0.27 ± 0.09	65.83 ± 1.85[d]	33.10 ± 1.90[d]

Percentage of cell death by apoptosis/necrosis of promastigote forms of ± SEM from three independent experiments in triplicate. [a] $P < 0.05$ vs. untreated control, [d] $P < 0.0001$ vs. untreated control, by Analysis of Variance (ANOVA) with the post hoc Tukey test, compared with the untreated control.

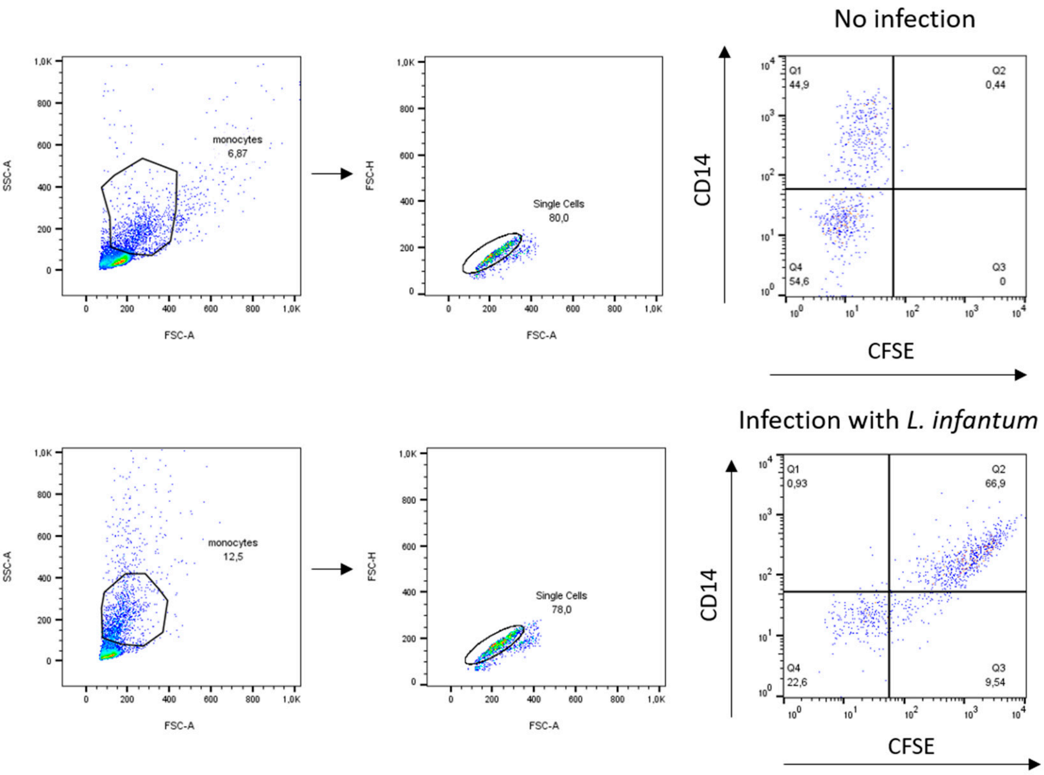

Figure S1. Analysis strategy of monocytes derived from human peripheral blood mononuclear cell (PBMC) using carboxyfluorescein diacetate succinimidyl ester (CFSE)-labeled *Leishmania infantum*. The PBMC cells were visualized on FSC vs. SSC and a wide gate was performed around the monocyte population, excluding most debris and lymphocytes. The cells were visualized on the FSC-H x FSC-A plot in single cells format. These cells were then visualized on the CD14 (PERCP) vs granularity (SSC) plot by selecting only the monocyte population. Finally, CD14+ cells were visualized on the CFSE vs. plot. CD14+, thus identifying monocytes infected by Leishmania sp.

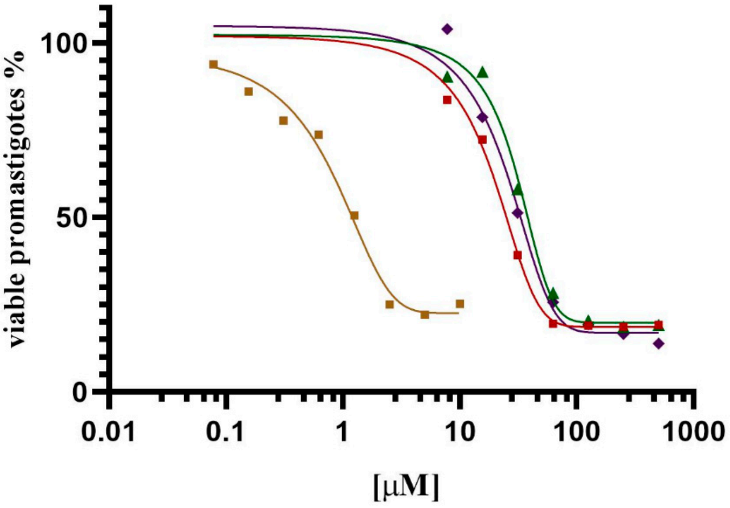

Figure S2. Dose-response curve fitting of promastigotes treated with LQOFG-2; LQOFG-6; LQOFG-7 and Amphotericin B.

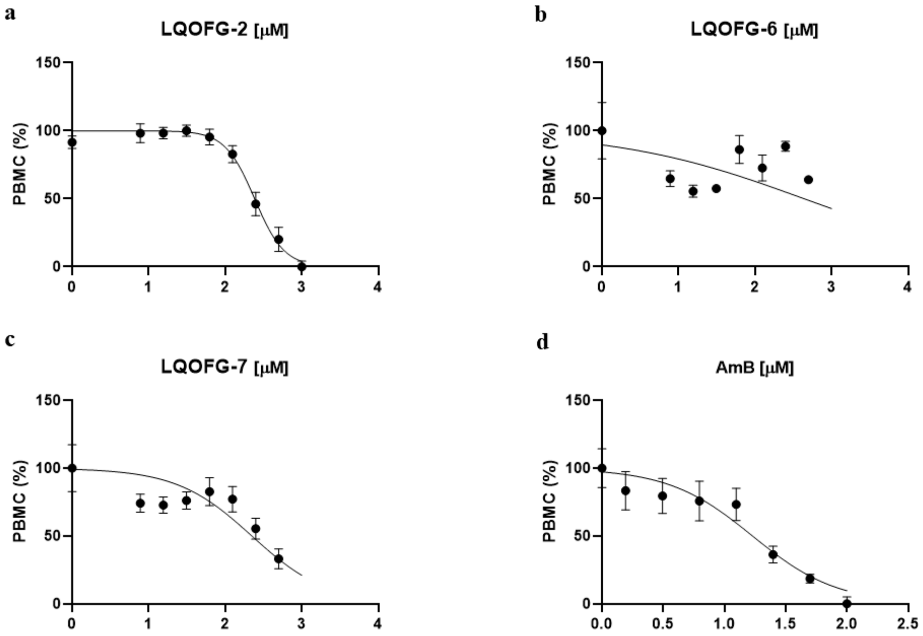

Figure S3. Dose-response curve fitting of PBMC cytotoxicity after LQOFG-2, LQOFG-6, LQOFG-7, and Amphotericin B treatment.
